# Whole genome sequence and a phylogenetic analysis of the G8P[14] group A rotavirus strain from roe deer

**DOI:** 10.1186/s12917-017-1280-4

**Published:** 2017-11-25

**Authors:** Urska Jamnikar-Ciglenecki, Urska Kuhar, Andrej Steyer, Andrej Kirbis

**Affiliations:** 1 Institute of Food safety, Feed and Environment, Veterinary Faculty, University of Ljubljana, Gerbičeva 60, 1000 Ljubljana, Slovenia; 2 Institute of Microbiology and Parasitology, Veterinary Faculty, University of Ljubljana, Gerbičeva 60, 1000 Ljubljana, Slovenia; 3 Institute of Microbiology and Immunology, Faculty of Medicine, University of Ljubljana, Zaloška 4, 1000 Ljubljana, Slovenia

**Keywords:** Group A rotavirus, Wildlife, Deer, Zoonotic transmission, Phylogenetic analysis, Next generation sequencing, NGS

## Abstract

**Background:**

Group A rotaviruses (RVA) are associated with acute gastroenteritis in children and in young domestic and wild animals. A RVA strain was detected from a roe deer for the first time during a survey of game animals in Slovenia in 2014. A further RVA strain (SLO/D110–15) was detected from a roe deer during 2015. The aim of this study was to provide a full genetic profile of the detected RVA strain from roe deer and to obtain additional information about zoonotic transmitted strains and potential reassortments between human rotavirus strains and zoonotic transmitted rotavirus strains. The next generation sequencing (NGS) analysis on Ion Torrent was performed and the whole genome sequence has been determined together with a phylogenetic analysis.

**Results:**

The whole genome sequence of SLO/D110–15 was obtained by NGS analyses on an IonTorrent platform. According to the genetic profile, the strain SLO/D110–15 clusters with the DS-1-like group and expresses the G8-P[14]-I2-R2-C2-M2-A3-N2-T6-E2-H3 genome constellation. Phylogenetic analysis shows that this roe deer G8P[14] strain is most closely related to RVA strains found in sheep, cattle and humans. A human RVA strain with the same genotype profile was detected in 2009 in Slovenia.

**Conclusions:**

The G8P[14] genotype has been found, for the first time, in deer, a newly described host from the order *Artiodactyla* for this RVA genotype. The finding of a rotavirus with the same genome segment constellation in humans indicates the possible zoonotic potential of this virus strain.

## Background

Group A rotaviruses (RVA) are members of the genus *Rotavirus* belonging to the highly diverse *Reoviridae* family, whose members are capable of infecting various host species (mammals, reptiles, fish, birds, fungi, plants and insects) [1]. Their genome consists of eleven double-stranded RNA segments that encode six structural proteins (VP1 to VP4, VP6 and VP7) and six non-structural proteins (NSP1–6) [2]. From a medical and veterinary perspective, RVA is the most important member of the genus and is associated with acute gastroenteritis in children and in young domestic and wild animals [2–4].

The RVA are classified according to the two outer capsid proteins VP7 and VP4, and at least 35 G-types and 50 P-types [5], respectively, have been characterized in humans and animals with more than 60 G-P combinations. In order to provide a better insight into RVA diversity and evolution, a new whole genome genotyping system was established and proposed by the Rotavirus Classification Working Group (RCWG) [6]. Under this classification system, the notation Gx–P[x]–Ix-Rx–Cx–Mx–Ax–Nx–Tx–Ex–Hx (“x” denotes the genotype number) has been used to represent the complete genotype constellation (VP7–VP4–VP6–VP1–VP2–VP3–NSP1–NSP2–NSP3–NSP4–NSP5 genes) of a RVA strain [7]. According to the genome segments’ genotype constellation, there are three main rotavirus genogroups, Wa-like (G1P[8]), DS-1-like (G2P[4]) and AU-1-like (G3P[9]), identified in humans and animals, respectively [8]. It was shown previously, that human Wa-like strains are related to porcine Wa-like strains and that human and bovine DS-1-like strains are also closely related. In addition, reports on bovine-like G6, G8 and G10 strains from the DS-1-like genogroup were frequently reported in human infections indicating the possible zoonotic transmission from animals to humans [9]. Whole genome sequencing is particularly important for the study of zoonotic transmitted strains and potential reassortments between human and animal RVA strains [10].

Rotaviruses have been reported in many ungulates, including deer [11], but only a few genomes of these RVA strains have been studied. Until recently, there have been no reports describing the genomes of RVA strains in deer. In our previous report [12], the first genome of the roe deer RVA strain was described as having the G6-P[15]-I2-R2-C2-M2-A3-N2-T6-E2-H3 genotype constellation. Later that year a red deer RVA strain from the USA with G8-P[1]-I2-R2-C2-M2-A3-N2-T6-E2-H3 was reported [13]. These two strains share the same bovine DS1-like genetic backbone but have different G/P combinations. Here we report a second roe deer RVA strain with the genotype constellation G8-P[14]-I2-R2-C2-M2-A3-N2-T6-E2-H3.

## Methods

### Sample collection and molecular detection of RVA

Initial findings from a survey conducted in 2014 and 2015 to screen certain game animals as a potential source of rotaviruses have previously been reported [12]. Screening of a further 15 samples from roe deer using specific RT-PCR and real-time RT-PCR [12] identified a further RVA-positive sample (SLO/D110–15). The sample was collected in October 2015, in Lahovče (hunting family Krvavec) from a one-year-old roe deer of appropriate weight for its age and exhibiting no specific clinical signs.

### RNA extraction, NGS and analysis of sequence reads

To determine the whole genome of the RVA strain SLO/D110–15, the sample was prepared for the NGS. Total RNA was extracted with Trizol reagent (Invitrogen, Carlsbad, USA) in combination with the RNeasy Mini Kit (Qiagen, Hilden, Germany), using the protocol with on-column DNase I digestion according to the manufacturer’s instructions. The RNA was used as the template for cDNA synthesis with the cDNA Synthesis System (Roche, Manheim, Germany) according to the Genome Sequencer Rapid RNA library protocol (Roche). The cDNA was fragmented with a Covaris M220 focused ultrasonicator, targeting peak fragments with lengths of 200–300 bp. The fragmented cDNA was used for library preparation using GeneRead DNA Library L Core Kit (Qiagen, Hilden, Germany). Purification and size selection of the library were performed with Ampure XP magnetic beads (Beckman Coulter, Brea, CA, USA). The library was quantified with the GeneRead Library Quant Kit (Qiagen, Hilden, Germany) and a Qubit 3.0 fluorometer (Thermo Fisher Scientific, Carlsbad, CA, USA). Emulsion PCR and enrichment were carried out using the Ion PGM™ Template OT2 200 Kit (Thermo Fisher Scientific, Carlsbad, CA, USA). The library was sequenced on the Ion PGM platform using the Ion PGM HiQ Sequencing Kit and Ion 314 Chip v2 (Thermo Fisher Scientific, Carlsbad, CA, USA). Sequenced reads were quality checked and trimmed using Ion Torrent Suite version 5.0.4 and assembled into contigs by de novo assembly, using the Genome Sequencer software version 2.9 (Roche, Basel, Switzerland). The contigs were compared to the GenBank non-redundant nucleotide database (BLASTn) to determine the contigs that represent the rotavirus genome segments. To eliminate assembly errors, all sequenced reads were mapped against the concatenated segments of the assembled RVA strain SLO/D110–15 genome with the Genome Sequencer software version 2.9 (Roche). The Geneious software suite v 9.0.5 (Biomatters LtD, Auckland, New Zeland) was used for visualization and final data analysis. To obtain the genomic constellation of the RVA strain, the RotaC v 2.0 online automated genotyping tool [14] was used to assign the genotype of each genome segment.

### Phylogenetic analysis of the genome segments

Selected sequences of RVA deposited in GenBank, with complete genome and genotype relevant to our strain, were used in the phylogenetic analyses. In addition, some of the most nearly identical sequences were added according to the BLAST search on each segment. The Slovenian RVA strain SI-2987/09, with genome profile G8-P[14]-I2-R2-C2-M2-A3-N2-T6-E2-H3 was also included in the genome nucleotide sequence identity analysis (Table 1). The strain was detected in a child with gastroenteritis during the RVA survey in 2009 described in the study by Steyer et al. [15]. As the strain SI-2987/09 was available only with partial nucleotide sequences of the genome segments, it was not included in the phylogenetic analysis.

**Table 1:** Genome genotype constellation of the 11 segments of RVA/Roe deer-wt/SLO/D110–15/G8P[14], the closest nucleotide identities from GenBank and identities shared with Slovenian RVA/Hu-wt/SVN/SI-2987/09/G8P[14] strain

Gene	Strain SLO/D110–15		Strains in the GenBank with the closest nucleotide identity			
	Genotype	Accession no.	Strain	Accession no.	Nt identity (%)	Host
VP7	G8	KY426808	OVR762	EF554153	96.9	Ovine
			SI-2987/09	KY972333	85.7^a^	Human
VP4	P[14]	KY426812	Tottori-SG	AB853893	94.7	Bovine
			SI-2987/09	KY972331	83.7^a^	Human
VP6	I2	KY426813	Tottori-SG	AB853894	94.1	Bovine
			SI-2987/09	KY972332	87.0^a^	Human
VP1	R2	KY426809	B10925	EF554115	94.8	Human
			SI-2987/09	KY972328	85.5^a^	Human
VP2	C2	KY426810	182-02	KU508381	92.3	Human
			SI-2987/09	KY972329	95.4^a^	Human
VP3	M2	KY426811	SI-R56	JX094030	98.6	Human
			SI-2987/09	KY972330	91.1^a^	Human
NSP1	A3	KY426803	NCDV	GU808570	97.2	Bovine
			SI-2987/09	KY972323	99.7^a^	Human
NSP2	N2	KY426804	1604	JN831216	96.6	Bovine
			SI-2987/09	KY972324	99.3^a^	Human
NSP3	T6	KY426805	UCD	GQ428138	97.8	Giraffe
			SI-2987/09	KY972325	93.1^a^	Human
NSP4	E2	KY426806	BEF06018	KU128901	97.8	Human
			SI-2987/09	KY972326	97.0^a^	Human
NSP5	H3	KY426813	BEF06018	KU128902	99.7	Human
			SI-2987/09	KY972327	95.1^a^	Human

^a^the identity scores were calculated with the partial nucleotide sequences of the rotavirus strain SI-2987/09

Nucleotides were aligned using ClustalW implemented in MEGA 7.0.21 [16]. Nucleotide identities were calculated according to the p-distances implemented in MEGA 7.0.21 [16]. Phylogenetic trees were constructed using the Maximum-likelihood method based on the Kimura-2 parameter model. Branch statistics were calculated by bootstrap analysis of 1000 replicates.

## Results

### NGS analyses and construction of the complete genome sequence

The complete genome sequence of all 11 segments of the RVA/roe deer-wt/SLO/D110–15/2015/G8P[14] strain was obtained using the Ion Torrent PGM platform. The nucleotide sequences of all eleven genome segments were deposited in GenBank and are available under the following accession numbers: KY426809 (VP1), KY426810 (VP2), KY426811 (VP3), KY426812 (VP4), KY426813 (VP6), KY426808 (VP7), KY426803 (NSP1), KY426804 (NSP2), KY426805 (NSP3), KY426806 (NSP4) and KY426807 (NSP5 and NSP6).

In total, 98,441 reads were generated and 25 large contigs (lengths >500 nt) obtained by de novo assembly. The BLAST search revealed that 11 of the contigs belong to the 11 RVA genome segments and constituted the complete genome of the RVA strain SLO/D110–15. Mapping against the concatenated segments of the RVA strain SLO/D110–15 genome resulted in 66,153 mapped reads (81.4% of all reads) with an average depth of 569.6 and average map length of 158 nt. Using the RotaC classification tool, the whole genome constellation of the RVA strain SLO/D110–15 was determined to be G8-P[14]-I2-R2-C2-M2-A3-N2-T6-E2-H3. The complete genotype constellation of the SLO/D110–15 strain was compared with those of RVA G8P[14] strains and other representative strains from humans and animals (Table 2). The genotype constellation of the SLO/D110–15 strain was identical to those of the two Italian human strains ITA/PR1300 and ITA/PR1973, and of the Slovenian human strain SI-2987/09, namely G8-P[14]-I2-R2-C2-M2-A3-N2-T6-E2-H3.

**Table 2:**
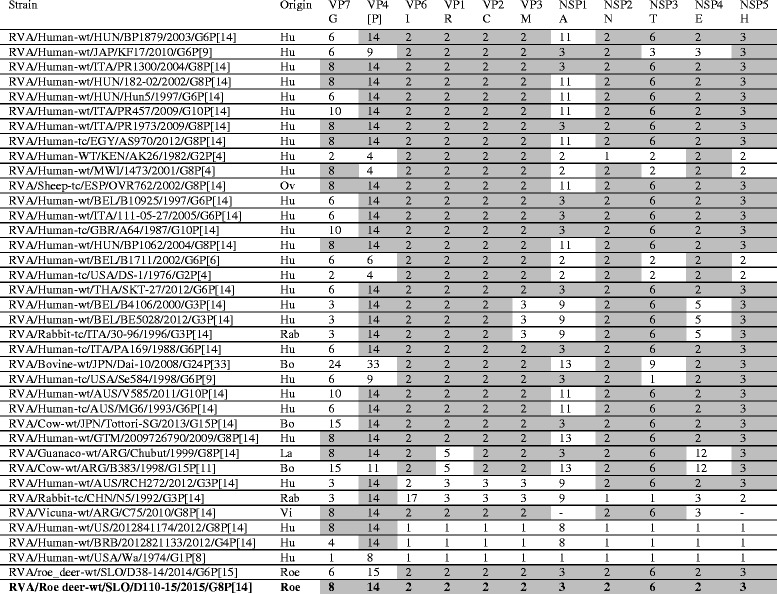
Comparison of the genotype constellation of Slovenian RVA roe deer SLO/D110–15 with other RVA complete genome sequences from GenBank

### Nucleotide sequence identity and phylogenetic analysis of the genome segments

p-Distances were calculated for each of the 11 segments of SLO/D110–15 and for selected strains from GenBank. The highest level of nucleotide identity, 99.7%, was observed on the NSP5 segment with strain BEL/BEF06018 and on the NSP1 segment with strain SI-2987/09. The lowest nucleotide identity was 92.3% and was observed on the VP2 segment with strain HUN/182–02 (Table 1). The roe deer SLO/D110–15 and human SI-2987/09 strains shared fewer than 90% nucleotide identities for partial sequences of structural genes VP1, VP6, VP7 and fragment VP8* of the VP4 gene, although sharing the same genotype. In contrast, the partial sequences of the non-structural protein genes shared higher degrees of identity, ranging from 93.1% to 99.7%.

**Fig. 1:**
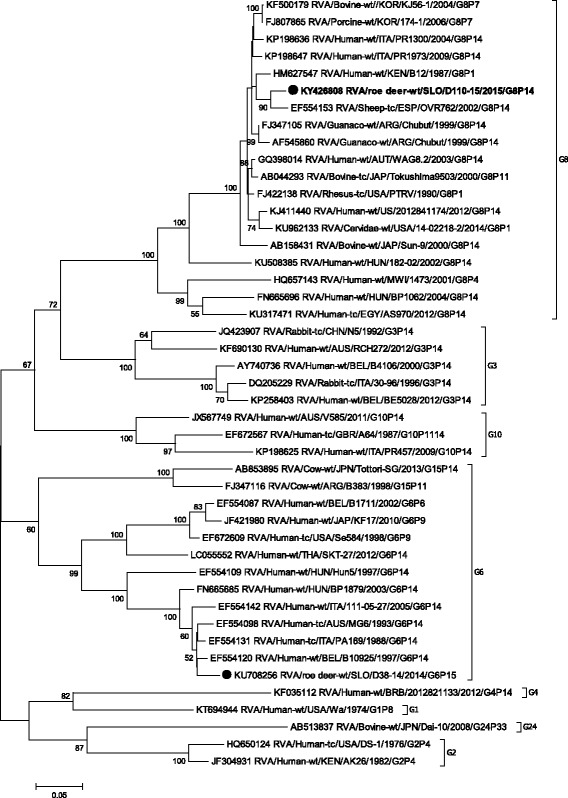
The Maximum likelihood phylogenetic tree on VP7 segment. Bootstrap values lower than 50 are not shown. The Slovenian roe deer strains SLO/D110–15 and SLO/D38–14 are marked with circle. Roe deer strain SLO/D110–15 is highlighted in bold

In the VP7 phylogenetic tree, the strain SLO/D110–15 was most closely related to the Spanish ovine strain ESP/OVR762, with a nucleotide identity of 96.6% (Table 1, Fig. 1) and, when compared to other G8 strains, the lowest nucleotide identity of 81.8%. Comparison of the VP7 segment with the other known roe deer sample, RVA SLO/D38–14, led to an identity of 85.8%.

**Fig. 2:**
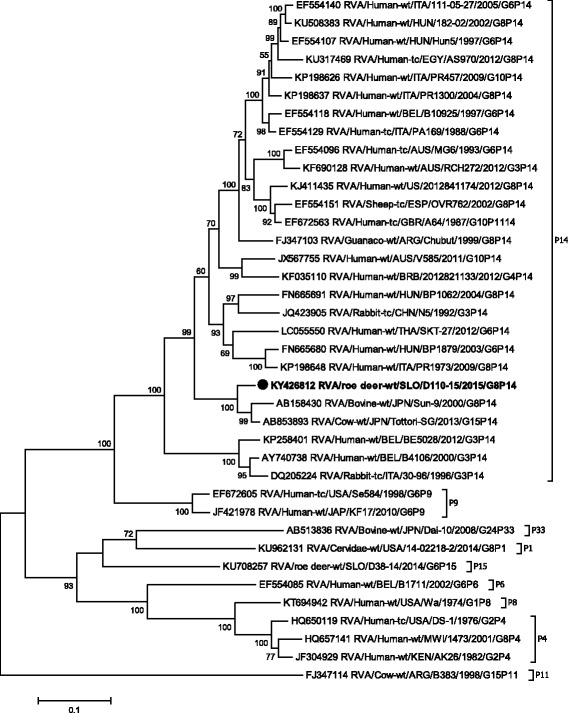
The Maximum likelihood phylogenetic tree on VP4 segment. Bootstrap values lower than 50 are not shown. The Slovenian roe deer strains SLO/D110–15 and SLO/D38–14 are marked with circle. Roe deer strain SLO/D110–15 is highlighted in bold

Phylogenetic analysis of the VP4 gene showed that the strain SLO/D110–15 formed a cluster with the Japanese P[14] bovine strains JPN/Tottori-SG and JPN/Sun9, with nucleotide identities of 94.8% and 93.2%, respectively (Table 1, Fig. 2). The lowest degree of nucleotide identity, when compared with other P[14] strains, was 80.9%. On the VP4 segment the nucleotide identity between roe deer RVA SLO/D110–15 and SLO/D38–14 was, as expected, low (67.3%), as they represent different P genotypes.

**Fig. 3:**
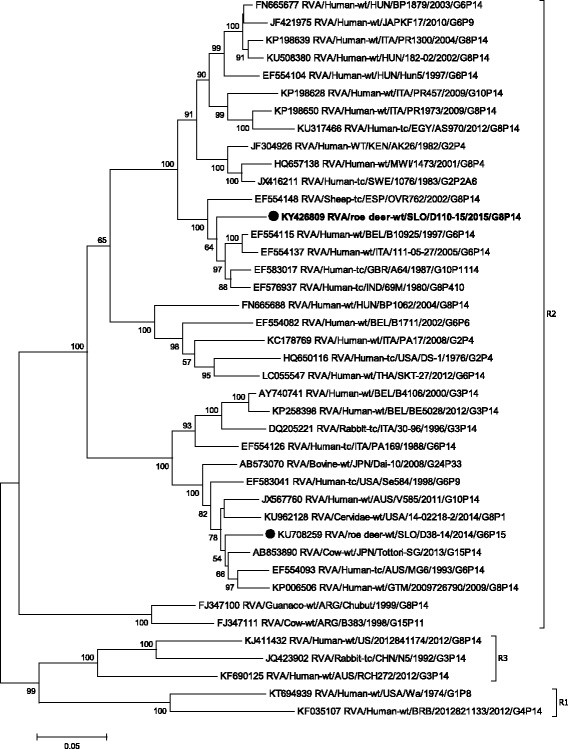
The Maximum likelihood phylogenetic tree on VP1 segment. Bootstrap values lower than 50 are not shown. The Slovenian roe deer strains SLO/D110–15 and SLO/D38–14 are marked with circle. Roe deer strain SLO/D110–15 is highlighted in bold

**Fig. 4:**
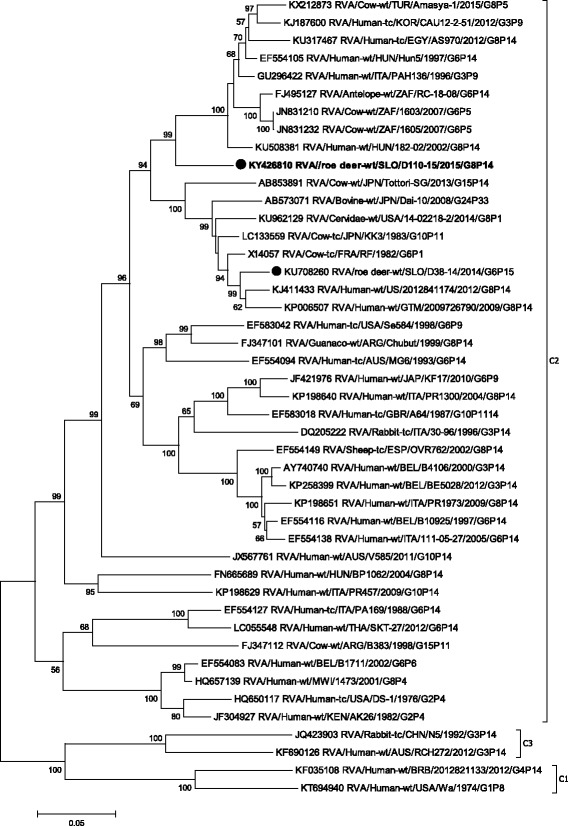
The Maximum likelihood phylogenetic tree on VP2 segment. Bootstrap values lower than 50 are not shown. The Slovenian roe deer strains SLO/D110–15 and SLO/D38–14 are marked with circle. Roe deer strain SLO/D110–15 is highlighted in bold

**Fig. 5:**
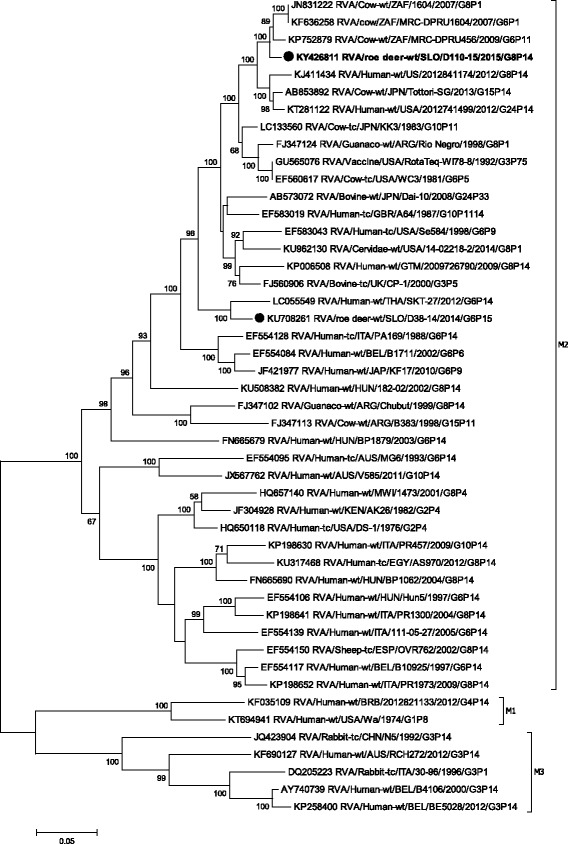
The Maximum likelihood phylogenetic tree on VP3 segment. Bootstrap values lower than 50 are not shown. The Slovenian roe deer strains SLO/D110–15 and SLO/D38–14 are marked with circle. Roe deer strain SLO/D110–15 is highlighted in bold

**Fig. 6:**
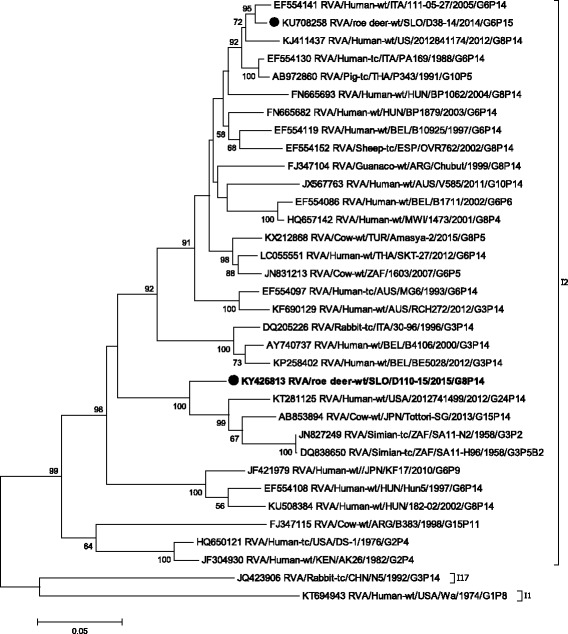
The Maximum likelihood phylogenetic tree on VP6 segment. Bootstrap values lower than 50 are not shown. The Slovenian roe deer strains SLO/D110–15 and SLO/D38–14 are marked with circle. Roe deer strain SLO/D110–15 is highlighted in bold

**Fig. 7:**
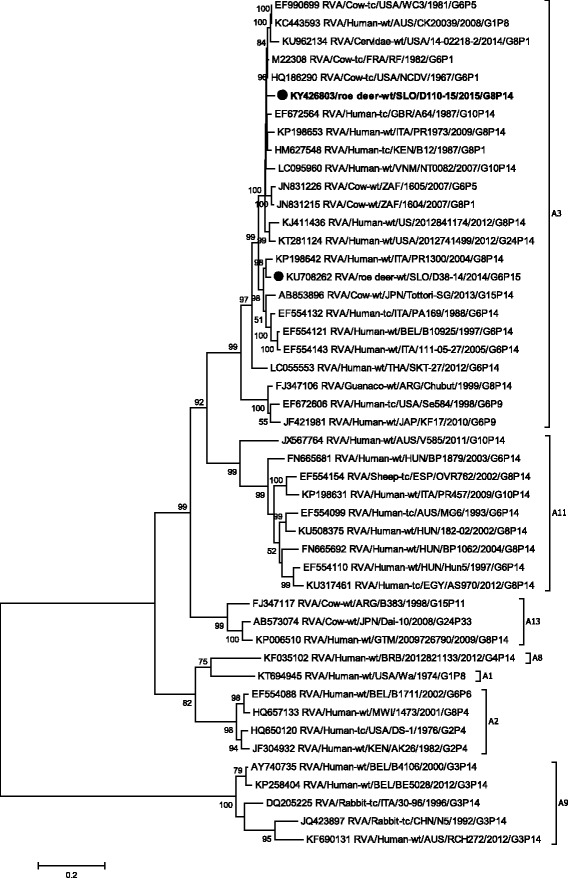
The Maximum likelihood phylogenetic tree on NSP1 segment. Bootstrap values lower than 50 are not shown. The Slovenian roe deer strains SLO/D110–15 and SLO/D38–14 are marked with circle. Roe deer strain SLO/D110–15 is highlighted in bold

**Fig. 8:**
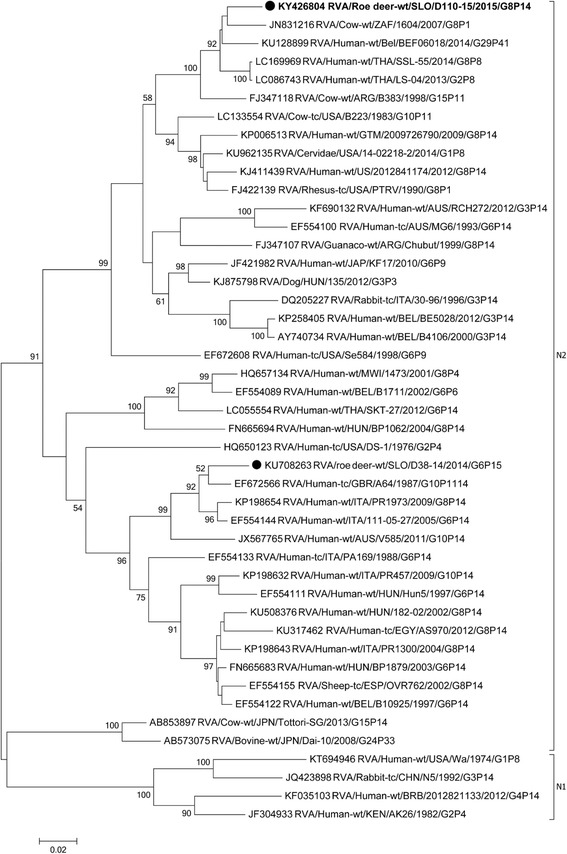
The Maximum likelihood phylogenetic tree on NSP2 segment. Bootstrap values lower than 50 are not shown. The Slovenian roe deer strains SLO/D110–15 and SLO/D38–14 are marked with circle. Roe deer strain SLO/D110–15 is highlighted in bold

**Fig. 9:**
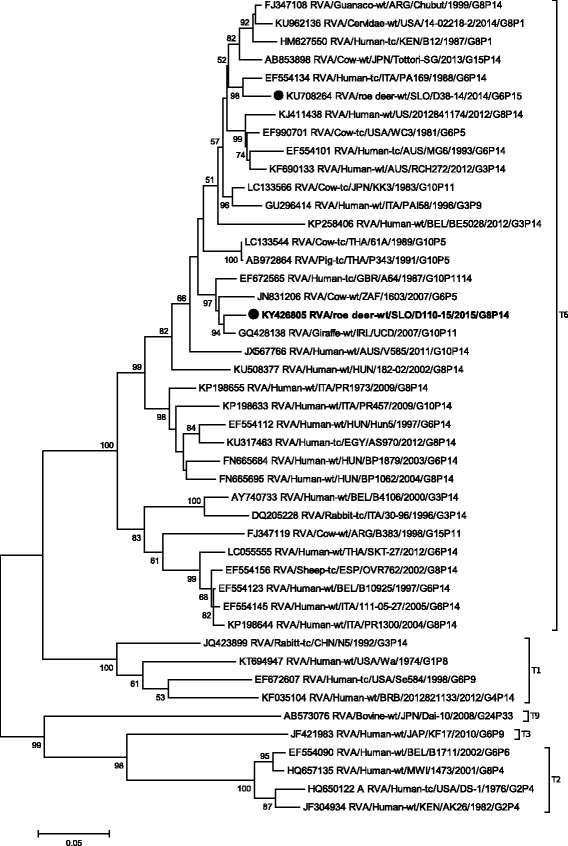
The Maximum likelihood phylogenetic tree on NSP3 segment. Bootstrap values lower than 50 are not shown. The Slovenian roe deer strains SLO/D110–15 and SLO/D38–14 are marked with circle. Roe deer strain SLO/D110–15 is highlighted in bold

**Fig. 10:**
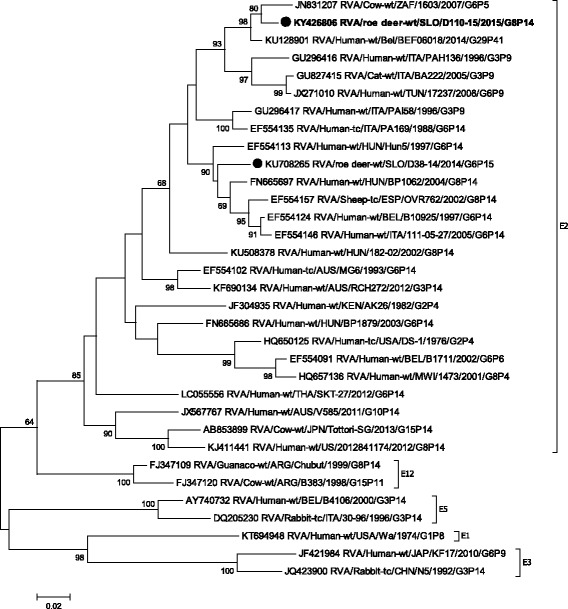
The Maximum likelihood phylogenetic tree on NSP4 segment. Bootstrap values lower than 50 are not shown. The Slovenian roe deer strains SLO/D110–15 and SLO/D38–14 are marked with circle. Roe deer strain SLO/D110–15 is highlighted in bold

**Fig. 11:**
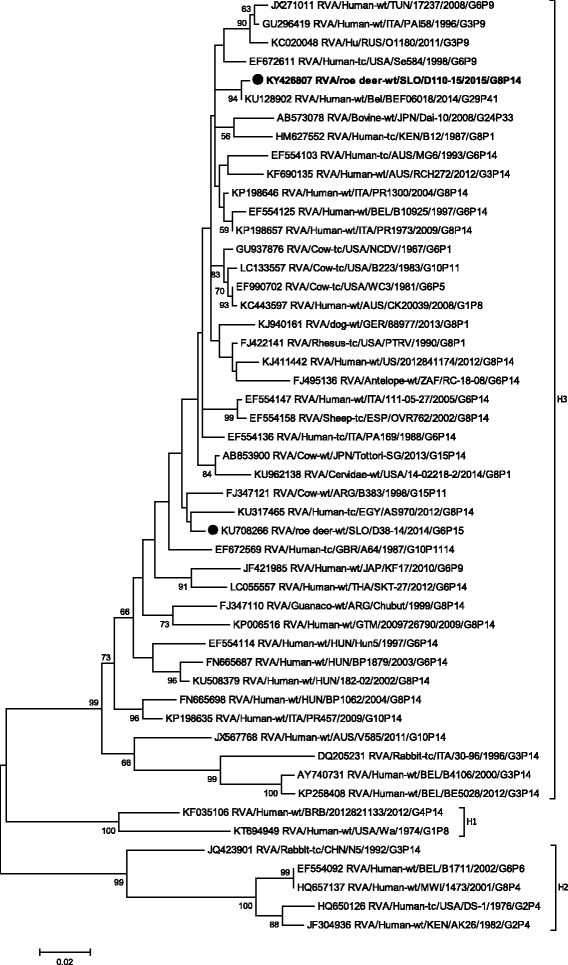
The Maximum likelihood phylogenetic tree on NSP5/NSP6 segment. Bootstrap values lower than 50 are not shown. The Slovenian roe deer strains SLO/D110–15 and SLO/D38–14 are marked with circle. Roe deer strain SLO/D110–15 is highlighted in bold

Phylogenetic analysis of VP1-VP3, VP6 and NSP1- NSP5 gene segments revealed that the SLO/D110–15 strain clusters with bovine and bovine-like RVA strains, sharing the same non-G/P genotype constellation (genetic backbone) I2-R2-C2-M2-A3-N2-T6-E2-H3 (Table 2) and with the highest degree of nucleotide identity in the range of 92.2% to 99.7% (Table 1, Figs. 3, 4, 5, 6, 7, 8, 9, 10 and 11).

When comparing segments from the backbone of roe deer SLO/D110–15 with those of roe deer SLO/D38–14, the highest degree of nucleotide identity ranged only from 85.8% to 96.8%, even though they share the same genetic backbone.

## Discussion

There are only two reports describing the complete genome constellation of RVA in deer. The first detection and complete genome characterization of a roe deer rotavirus was in 2015 [12]. Here, we describe the second detection of RVA in roe deer and the first RVA strain with the G8-P[14]-I2-R2-C2-M2-A3-N2-T6-E2-H3 genotype constellation. Important insights into the complete genetic makeup of a deer rotavirus strain are provided in this report and consequently new knowledge about the host range for this RVA genotype, together with their strain diversity.

The sequencing result of the SLO/D110–15 roe deer sample investigated in this study revealed a large number of RVA sequencing reads (81.4% of all sequencing reads), using an unbiased protocol for sample and library preparation, and with no observed clinical signs in the respective animal. The NGS analyses show that, unlike the first positive roe deer sample collected in 2014 in Slovenia, SLO/D38–14, that belongs to the G6P[15] genotype, the roe deer sample SLO/D110–15 belongs to the G8P[14] genotype. They share from 67.3% to 96.8% genome segment nucleotide identity.

The strain SLO/D110–15 has the typical bovine DS1-like genetic backbone found in cattle and other animals from the order *Artiodactyla* together with a G/P combination also found in zoonotic human RVA strains. Detailed phylogenetic analysis of the 11 genome segments revealed the closest relatedness of the SLO/D110–15 strain to RVA strains having the bovine-like genotype constellation from humans and animals.

RVA strains of the G8P[14] genotype, combined with the bovine DS1-like genetic backbone, are detected sporadically in cattle, sheep, guanaco, vicuna and in humans [17–23]. It was suggested that the P[14] genotypes are less virulent for the ruminant host species, and thus more probably shed by animals with subclinical infections [20]. This was supported by our finding that the SLO/D110–15 was detected in an animal without evident clinical signs. However, the real pathogenic potential of these rotaviruses still has to be explored. Virus isolation in MA104 cell line was attempted for the strain SLO/D110–15 but was not successful, even after three passages.

The original source of RVA strains with G8P[14] genotype most probably includes multiple human to animal and animal to human transmission events [24]. It has been proposed that human P[14] strains are derived from interspecies transmission of RVA from humans and ungulates [17]. Other studies suggest zoonotic transmissions of the G8P[14] RVA strains with the bovine DS1-like genetic backbone [20]. Two human strains from Italy, PR1300 and PR1973 have a full genome constellation identical to that of our roe deer strain (G8-P[14]-I2-R2-C2-M2-A3-N2-T6-E2-H3) [19]. For these two human strains it was suggested that they are zoonotic and transmitted to humans from an animal belonging to the order *Artiodactyla* [19]. In addition, one G8P[14] strain with a genotype constellation identical to that of the roe deer strain, was observed in a child with gastroenteritis during the RVA survey in Slovenia in 2009 [15]. Although the genome segment sequences of this RVA human strain are not complete, it was shown that human and roe deer strains from Slovenia are not closely related as the VP8* fragment and partial VP7 nucleotide sequence identities were both less than 90%. Relatively low identities between these two strains were shown also for other genes coding for structural proteins. For genes coding non-structural proteins the identities were much higher, indicating the reassortant nature of G8P[14], probably as a result of circulating in different hosts. Solving the riddle of the evolutionary path of G8P[14] strains requires the analysis of many more strains. Surveillance of the RVA in animals and humans should be continued to gain a clearer molecular and epidemiological history of zoonotic RVA strains.

## Conclusions

The G8P[14] genotype has been found, for the first time, in deer, a newly described host from the order *Artiodactyla* for this RVA genotype. The finding of a RVA strain with the same genome segment constellation in humans indicates the possible zoonotic potential of this virus strain.

## Abbreviations

NGS: next generation sequencing technology
RCWG: Rotavirus Classification Working Group
RVA: Group A rotaviruses
